# Clinical and radiologic rebound after discontinuation of natalizumab therapy in a highly active multiple sclerosis patient was not halted by dimethyl-fumarate: a case report

**DOI:** 10.1186/s12883-015-0512-0

**Published:** 2015-12-07

**Authors:** Francesco Patti, Carmela Leone, Mario Zappia

**Affiliations:** Department of Medical Sciences, Surgicals and Advanced Technologies G.F. Ingrassia, Section of Neuroscience, University of Catania, Via Santa Sofia 78, 95123 Catania, Italy

**Keywords:** Multiple sclerosis, Natalizumab discontinuation, Dimethyl-fumarate, Clinical rebound, Radiologic activity, Switching therapy

## Abstract

**Background:**

The evidence on the use of the oral dimethyl-fumarate after the discontinuation of treatment with natalizumab in people with Multiple Sclerosis is still little. Natalizumab discontinuation may induce the recurrence or rebound of the clinical and neuroradiological disease activity. Currently no therapeutic approach has been established to abolish disease reactivation and rebound after natalizumab interruption.

**Case Presentation:**

We describe a case of a 21-year-old woman affected from a highly active relapsing-remitting Multiple Sclerosis who developed a clinical and radiological rebound 5 months after the last infusion of natalizumab, while she was being treated with dimethyl-fumarate 240 mg twice daily. She had received a bridge “therapy” with Cyclophosphamide before staring dimethyl-fumarate.

**Conclusion:**

We report on this case to stimulate further research to establish whether new current and future drugs available for multiple sclerosis are able to halt the disease rebound after the natalizumab interruption.

## Background

Natalizumab (NAT), a specific a4-integrin antagonist blocking lymphocytes transmigration across the blood–brain barrier, is a second-line treatment of active relapsing-remitting (RR) multiple sclerosis (MS). The benefit of reduction in relapse rate, disability progression and Magnetic Resonance Imaging (MRI) lesions load has to be weighed against the risks of adverse events [1]. Which is mainly related to the rare but serious progressive multifocal leukoencephalopathy (PML); longer treatment duration increases the risk of this adverse event [2, 3]. After NAT discontinuation, MRI and clinical disease activity gradually return to the pre-treatment levels [2], sometimes even a rebound with a flare-up to a level beyond the pre-NAT treatment level was reported [4–6]. There are no available randomized controlled trials or established guidelines on “what to do after NAT therapy”. The RESTORE study showed that disease activity began 12-weeks after NAT-discontinuation and occurred regardless of following “drug holiday”, “bridge” therapy or “switch” to an alternative treatment with either glatimarer acetate or interferons [7]. On the contrary, those who continued NAT did not show MRI evidence of new disease activity, suggesting that only NAT can stop the rebound due to NAT-interruption. However, in that study [7], new switching options which are available either currently or in the near future, such as fingolimod, dimethyl-fumarate (DMF), teriflunomide and alemtuzumab were not included. Furthermore, other observational studies were conducted to investigate the effect of fingolimod in preventing disease reactivation after NAT discontinuation [8–12]. Among them, five studies showed clearly that early initiation of fingolimod (less than 2 or 3 months after discontinuing NAT) decreases the probability of disease re-activation [8, 10–13], highlighting the importance of an early treatment after NAT withdrawal. Iaffaldano et al. showed a superiority of fingolimod in comparison to interferon beta/glatiramer acetate in controlling disease reactivation after NAT discontinuation in a large sample of real life setting [13]. Preliminary evidences showed both positive [14, 15], and negative effect of DMF on minimizing disease activity in persons with MS switching from NAT [16].

To summarize, data about MS rebound occurrences in patients treated with DMF after NAT-discontinuation are not available in literature.

## Case presentation

**Fig. 1:**
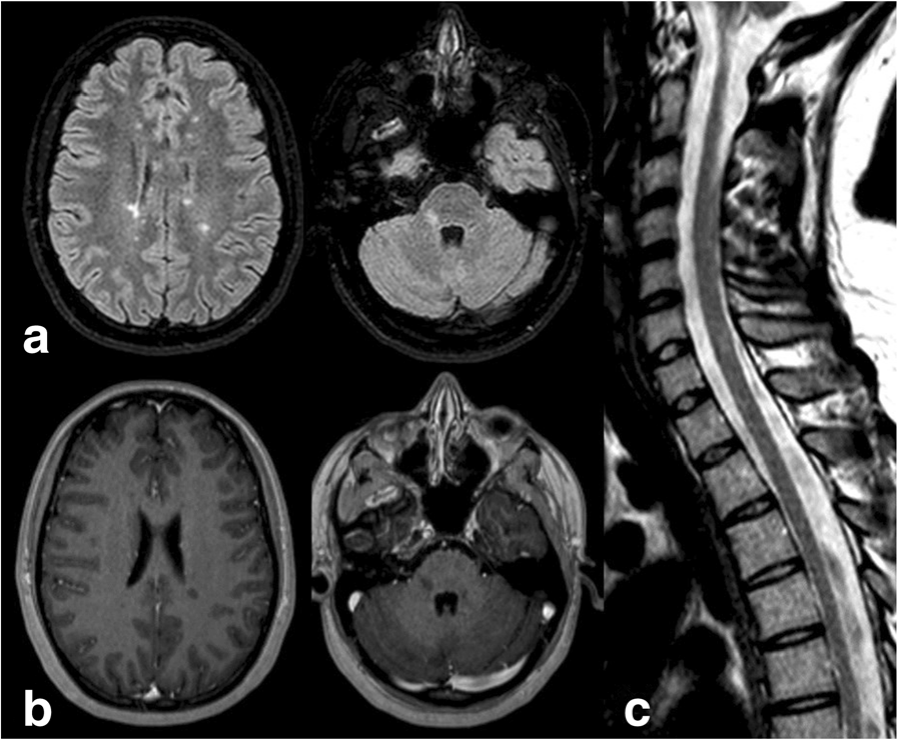
Axial (**a**) T2-weighted FLAIR MRI under stable clinical conditions, showing several supratentorial lesions; axial (**b**) T1-weighted images showing no lesions with ring gadolinium-enhancement, (**c**) sagittal T2-weighted FLAIR MRI showing two spinal cord lesions

We report the case of a 21-year-old woman who in August 2011 was diagnosed with RRMS. She had no previous relevant medical conditions, nor family history of immune diseases. The disease onset was on May 2011 with acute cerebellar-related balance problems and spontaneously recovered after 3 weeks (EDSS 1.5). Shortly after the diagnosis, in October 2011, she was enrolled in the DECIDE study (double blind randomized controlled trial with IFN-beta 1a and Daclizumab 150 mg (DAC-HYP). However, she withdrew at early stages from this study (July 2012) due to the occurrence of two MS-relapses, both characterized by bi-ocular diplopia and blurred vision (EDSS 3.0). She completely recovered from this relapse after high dose of i.v. steroids. An MRI scan performed on May 2012 showed important radiological disease activity in the brain (25 T2-weighted and 7 T1-weighted/gadolinium-enhanced lesions) as well as in the spine (9 T2-weitghed and 2 T1-weighted/gadolinium-enhanced lesions). In August 2012, she started i.v. NAT 300 mg every 28 days. She was seropositive for JC-virus antibody status. During the 2 years of NAT-treatment, she was free of clinical activity and had a stable disability level (EDSS 1.5). She also underwent serial brain and spine MRI scans every 6 months, which showed no radiological disease activity as shown in Fig. 1. However, after 24 NAT-infusions (August 2014), administration of NAT was interrupted due to the risk of PML.

**Fig. 2:**
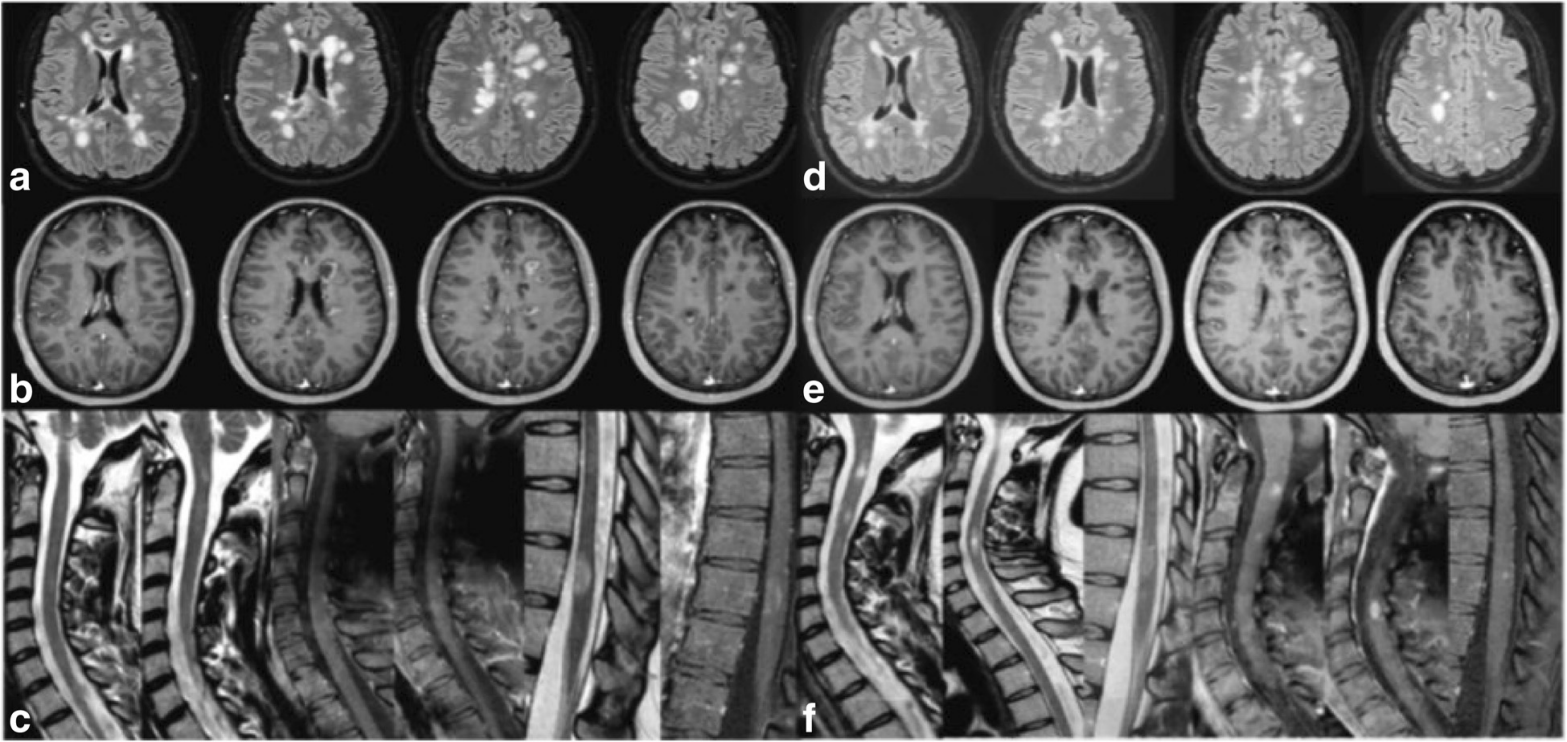
Axial (**a**) T2-weighted FLAIR MRI under clinical relapse showing multiple new supratentorial lesions; axial (**b**) T1-weighted images showing lesions with ring gadolinium-enhancement, (**c**) sagittal T2-weighted FLAIR MRI showing different spinal cord lesions, one of them with ring gadolinium-enhancement on T1-SPIR weighted image. (**d**), (**e**), (**f**) are the same images repeated after 40 days from high dose of steroids i.v.

A single pulse of i.v. cyclophosphamide 750 mg was administered in September 2014. Following this, in October 2014 she was switched to DMF, starting with 120 mg twice-a-day for the first 2 weeks, and then switching to DMF 240 mg twice-a-day. Shortly, after the change of dosage, she started to complain of adverse effects in the region of gastro-intestine, which was mainly characterized by nausea and weight-loss – by January 2015, she lost 15 kg. On 15/01/2015, she started to complain of new symptoms characterized by lower limb weakness, dizziness and gait ataxia, followed by bilateral blurred vision, in the absence of fever and other infection signs. When she arrived to our clinic, the neurological examination revealed an EDSS of 6.0 since the patient was able to walk only with a unilateral support for about 100 m. A new brain MRI showed massive progression in T2 lesions and lesions with ring gadolinium-enhancement numbers (>20 gadolinium-enhanced lesions, Fig. 2). A 5-day steroid course was then administered intravenously with great clinical benefit. The MRI performed 1 month later showed a reduction of the number of gadolinium-enhanced lesions in the brain. After one additional month, she recovered completely from the relapse and the neurological examination showed an EDSS of 1.5. On 24/02/2015 she re-started the treatment with NAT. The new JC-virus antibody status index was three.

The young MS patient reported in this case had a severe clinical and radiological rebound after NAT-interruption, although she was being treated with DMF, which in turn was started as after a “bridge” therapy with cyclophosphamide.

DMF, an oral treatment for RRMS with immunomodulatory and cytoprotective effects [17], was not able to stop the clinical and MRI activity after NAT-interruption. Recent preliminary results on the effect of DMF on minimizing disease activity related to NAT-interruption have been reported with inconclusive results. Two studies reported on beneficial effects [14, 15], and one showed higher disease activity (frequency of patients with relapse) during DMF treatment period compared with NAT period treatment [16]. However, no rebound activity of MS has been shown so far. The AFFIRM, SENTINEL and GLANCE studies showed no rebound [2], but the RESTORE reported a high rate of disease activity after NAT-interruption despite a new drug was started [7]. Since this study proved the superiority of continuing NAT in comparison with its interruption despite switching to first-line disease modifying drugs or methylprednisolone, we decided to restart therapy with NAT. Despite her risk of PML, which was likely higher due to the single pulse of immunosuppressant we administered as a bridge therapy. We however started an accurate and strict monitoring of clinical and radiological activity including MRI scans every 3–4 months.

Yet, the best treatment of patients who withdraw from NAT independently from the interruption reasons does not exist. Several strategies applied in clinical practice, such as “bridge” therapy with methylprednisolone, switching to glatimarer acetate or interferon-beta are likely not effective [18]. However, more promising results came from several small [8, 10] and large retrospective [11, 12] and prospective [13] studies with fingolimod (FTY), but long-term data about its efficacy and safety after NAT are urgently needed. Finally we cannot exclude that the single pulse of cyclophosphamide we used as “bridge” therapy to DMF might have delayed the occurrence of a rebound of another 3 months.

## Conclusions

To summarize, this is the first case of MS rebound after switching from NAT to DMF, independently from the single pulse of cyclophosphamide. Yet, the best therapeutic approach to abolish the disease reactivation after the NAT-discontinuation needs to be found. Considering the narrow time window for the diminishing clinical and biological effects of NAT, we suggest to investigate in depth whether starting the new MS therapy together with the NAT (during i.e. the last three months of the NAT treatment) may be effective.

## Ethics

Any ethics was required for this case presentation, since we are reporting on clinical individual data of a patient followed in our Neurologic Clinic in Catania, Italy.

## Consent to publish

A written informed consent for data publication was obtained from the patient.

## Availability of data and materials

The source documents/files supporting our data are stored in the archive of our clinic and in the iMed© software (iMed, Merck Serono SA - Geneva).

## Abbreviations

NAT: natalizumab
RR: relapsing-remitting
MS: multiple sclerosis
DMF: dimethyl-fumarate
PML: progressive multifocal leukoencephalopathy
